# Implementing video group consultations in general practice during COVID-19: a qualitative study

**DOI:** 10.3399/BJGP.2021.0673

**Published:** 2022-05-31

**Authors:** Chrysanthi Papoutsi, Sara Shaw, Trisha Greenhalgh

**Affiliations:** Nuffield Department of Primary Care Health Sciences, University of Oxford, Oxford.; Nuffield Department of Primary Care Health Sciences, University of Oxford, Oxford.; Nuffield Department of Primary Care Health Sciences, University of Oxford, Oxford.

**Keywords:** digital primary care, general practice, group consultations, remote consultation, shared medical appointments

## Abstract

**Background:**

Group consultations have been gaining ground as a novel approach to service delivery. When in-person care was restricted owing to COVID-19, general practice staff began delivering group consultations remotely over video.

**Aim:**

To examine how multiple interacting influences underpinned implementation and delivery of video group consultations (VGCs).

**Design and setting:**

Qualitative study in general practice in England.

**Method:**

a) 32 semi-structured interviews with patients, clinical, and non-clinical staff (from eight GP surgeries in total), NHS policymakers and programme managers, and other stakeholders; b) observation in relevant training and operational meetings; and c) three co-design workshops (21 participants). Thematic analysis was informed by the Planning and Evaluating Remote Consulting Services (PERCS) framework.

**Results:**

In the first year of the pandemic, VGCs focused on supporting those with long-term conditions or other shared health and social needs. Most patients welcomed clinical and peer input, and the opportunity to access their practice remotely during lockdown. However, not everyone agreed to engage in group-based care or was able to access IT equipment. At practice level, significant work was needed to deliver VGCs, such as setting up the digital infrastructure, gaining team buy-in, developing new patient-facing online facilitation roles, managing background operational processes, protecting online confidentiality, and ensuring professional indemnity cover. Training provided nationally was seen as instrumental in capacity building for VGC implementation.

**Conclusion:**

Small scale VGC implementation addressed unmet need during the pandemic. However, embedding VGCs in routine care requires rethinking of operational, infrastructural, and clinical processes. Additional research on costs and benefits at service and patient level is needed.

## INTRODUCTION

In the UK there has been a strong policy push towards ‘digital first’ or what has recently been termed ‘augmented’ primary care, including online and video consultations.^1^^–^^4^ This drive has been substantially accelerated by the COVID-19 pandemic, with practices adopting triage models to manage patient contact, in the midst of an unprecedented workload and service disruption.^5^^–^^7^

Before the pandemic, group consultations (or shared medical appointments) started to gain traction as a potential means for managing rising demand in general practice.^2^^,^^8^^–^^12^ Combining clinical care with elements of group education and peer support, group consultations have been delivered in a variety of formats for patients with long-term conditions or shared health concerns.^8^^,^^13^

Research suggests group consultations have the potential to improve patient health and wellbeing, support job satisfaction, and contribute to service improvement.^14^ Studies in diabetes show improvements in glycaemic control, problem-solving ability, quality of life, and reduced time commitment for clinicians compared with one-to-one consultations,^15^^–^^18^ as well as positive patient experiences and better engagement with self-management.^19^ It is unclear how these findings in highly selected populations will transfer if group consultations are delivered at scale. It has also been suggested that face-to-face group consultations can contribute to patient-centred care and job satisfaction for healthcare staff,^12^^,^^20^ but local implementation can be challenging.^13^^,^^19^

In the context of COVID-19 restrictions, some GP practices in the UK started using video platforms to deliver group-based care remotely.^21^^–^^24^ They were supported by a training programme commissioned by NHS England and NHS Improvement (NHSE/I) that included a detailed toolkit, opportunities for inter-organisational learning and IT support, and an e-learning package.^25^ Originally designed for face-to-face group consultations, the programme was repurposed at the start of the pandemic to focus on video group consultations (VGCs). Approximately 1000 clinical and non-clinical staff took part in training over a period of 8 months, according to the training provider.

This study primarily focused on different approaches to VGC delivery and practice-level implementation challenges, asking:

- What types and formats of remote group-based care have been delivered in the context of COVID-19 in English general practice?
- How did multiple interacting influences shape VGC implementation?
- What were the views and experiences of patients and staff?
- What operational work and organisational adaptations were needed to deliver VGCs for different conditions and population groups?

**Table:** How this fits in

Previous research on face-to-face group consultations points to improvements in outcomes, but also implementation challenges in practice. Yet, little is known on how video group consultations (VGCs) delivered remotely may be implemented. In this qualitative study in general practice, most patients and staff expressed largely positive experiences with this new model of care during COVID-19 restrictions. Additional work was needed to support caring relationships at a distance, enable IT and online facilitation skills, align with remote care practices in the crisis context, and take account of digital inclusion.

## METHOD

Qualitative research was conducted with eight early adopter general practices in England, as part of a research programme on video consulting during the pandemic, with data collection taking place from April 2020 to April 2021.^26^^,^^27^

### Data collection

The study included 32 semi-structured interviews with:

- 15 NHS staff from eight practices (GPs, nurses, receptionists, a pharmacist, practice managers, a physiotherapist, and an IT officer);
- five patients who had participated in or declined VGCs;
- five national level policymakers and programme managers; and
- three participants from VGC training providers and the IT industry.

To gain in-depth understanding at different implementation stages, two of the eight GP practices were followed over 12 months, with key staff members interviewed at least twice (four repeat interviews) and having informal calls and email exchanges (Box 1).

**Box 1.** Group-based care delivery in two general practices where longitudinal data collection was carried out

	**Location**	**Practice characteristics**	**Group-based care**
**Site A**	Urban, South East (England)	Large practice (15 000 patients), multi-ethnic population in deprived area	Pharmacist leading diabetes VGCs. Initial set-up was slow, but at the time of writing (June 2021) the practice had involved 90 patients. Significant experience with face-to-face group consultations pre-pandemic (600 patients in 2 years).
**Site B**	Semi-rural, North West (England)	Medium-size practice (6000 patients), low levels of ethnic diversity and deprivation.	GP nurse leading VGCs every 2–3 weeks for patients with asthma, diabetes, COPD, and cancer (total = 40 patients in 8 VGCs). Initially started as a face-to-face programme but quickly became digital owing to COVID-19.

*COPD = chronic obstructive pulmonary disease. VGC = video group consultation.*

Interviews lasted 20–100 minutes (via MS Teams or phone) and the majority (*n* = 24/32) were audiorecorded and transcribed (with consent). In the rest of the interviews contemporaneous notes were taken, including verbatim quotes. Purposive and snowball sampling were used, recruiting participants through training providers, the NHSE/I programme, and interviewee recommendations. The interview agenda included questions on the reasons for setting up VGCs, how they were received by patients and staff, what worked well and less well in VGC implementation and delivery, how this model of care influenced the clinical relationship, and what organisational work was needed to sustain group-based care.

Online VGC training (*n* = 2), policy and programme meetings (*n* = 7), and industry meetings (*n* = 2) were observed. These lasted between 1–1.5 hours and included a range of 6–17 attendees. The authors often participated in discussions as part of these meetings to provide feedback and reflect on findings, and took contemporaneous notes that informed iterative data analysis. Combined with review of documents (for example, toolkits, email communication, and NHS Future Forums), informal exchanges contributed to data interpretation. Data were also analysed from a patient feedback survey distributed after VGC sessions in the two practices, using findings to inform the qualitative research.

With industry and co-design partners, three workshops (1.5 hours each) were held with patients, NHS staff, and NHSE/I programme partners (21 participants in total), to better understand what a good software platform might look like for VGCs and what equity and digital exclusion challenges VGCs surfaced. Detailed notes were taken and outputs retained (for example, digital post-it notes, diagrams, and conceptual prototypes) for analysis. Patient and public involvement (PPI) contributors supported the study, including through two group discussions and other ad hoc exchanges.

### Data analysis

**Figure 1.**
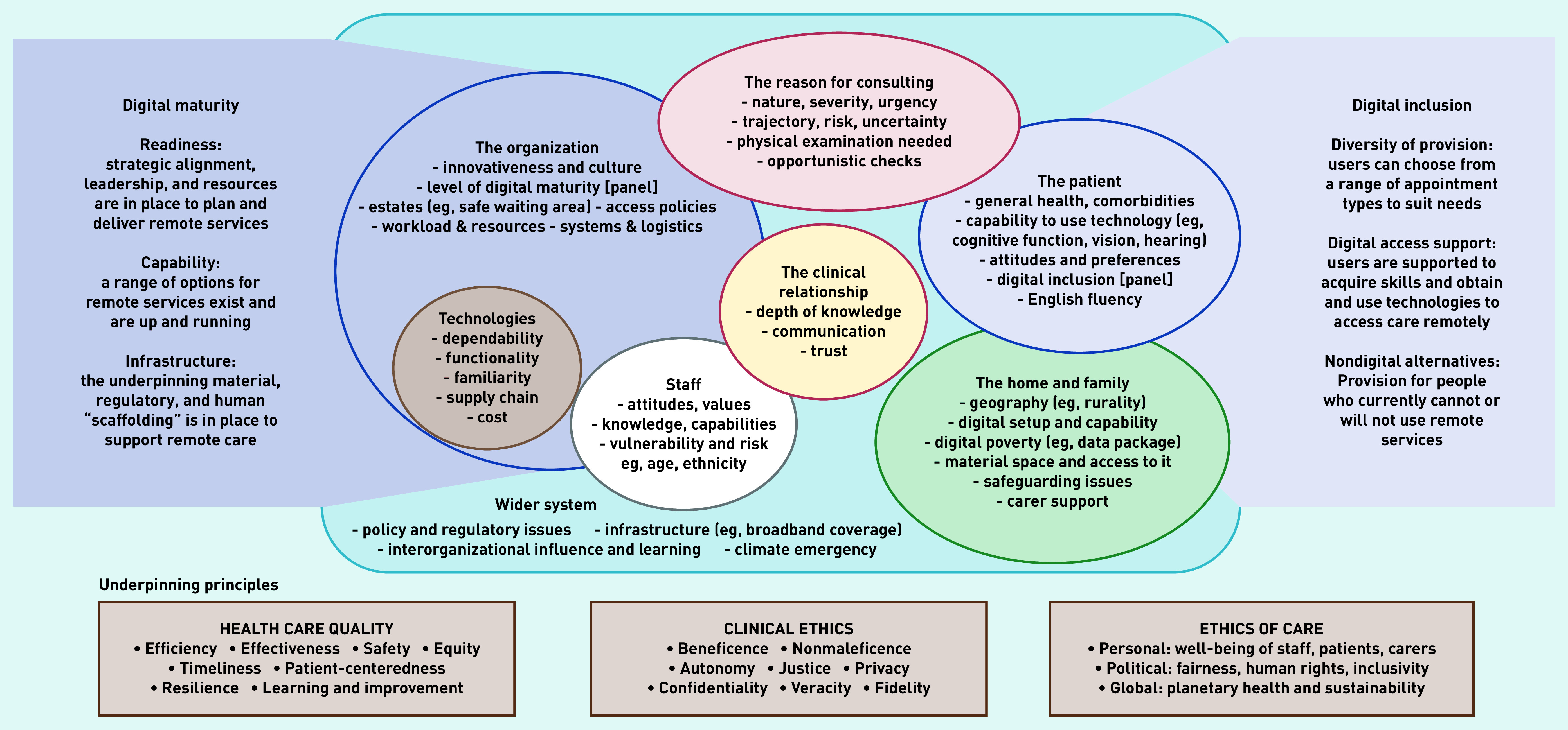
***PERCS (Planning and Evaluating Remote Consultation Services) framework. Reproduced under Creative Commons Licence form.*** *^26^*

Thematic analysis evolved in parallel with fieldwork, and was informed by the Planning and Evaluating Remote Consultation Services (PERCS) framework^26^ on multiple interacting influences in uptake of remote care, including the clinical relationship, staff attitudes/capabilities, and organisational capacity (Figure 1). The data were coded inductively and deductively, based on PERCS but also extending this to cover issues relevant to VGCs. Subsequently, narrative summaries were developed that bridged across PERCS domains to surface interdependencies (that is, the overlaps between domains illustrated in Figure 1) and address the research questions.

The analysis also drew on an understanding of crisis-as-process (rather than crisis-as-event)^28^ and a dynamic view of relational coordination to foreground the interdependent and complex nature of VGC organising.^29^ The first author led on the analysis and discussed findings with the rest of the research team during analysis and synthesis.

## RESULTS

### Diversity of formats and purposes

Interviewees described delivery of remote group-based care in different formats (Box 2). The term VGC was used interchangeably regardless of whether individually-focused clinical consultations took place in the group setting (which is usually the key prerequisite for a session to be described as a ‘group consultation’ according to published literature and the NHSE/I-commissioned training).

**Box 2.** Remote group-based care formats

**VGC type**	**Characteristics**
Clinical	One-to-one clinical discussion on test results, medication, and self-management in the group setting, combined with peer support and discussion
Educational	Focusing on self-management or ‘healthy living’ topics, with little or no individual clinical input
Informational	Webinar format rather than clinical consultation, usually involving a large number of attendees
Mixed	Combining elements from the first three types

*VGC = video group consultation.*

All formats included *formal* components, such as structured annual reviews for long-term conditions, and *informal* components, such as patient-driven, open discussion. They were delivered as scheduled sessions where patients with a relevant condition could book into standard slots or in a *targeted* way, inviting patients with specific clinical and/or social needs such as vulnerable new families (even across different practices). Patient participation was either *periodic*, to match annual or 6-monthly review requirements for chronic conditions, or more *regular*, with patients joining multiple, sometimes consecutive, sessions (with the same or different groups). Staff who were interviewed delivered VGCs primarily using clinical or mixed formats across a range of conditions and needs, including for patients with diabetes, asthma, chronic obstructive pulmonary disease (COPD), cancer (acute treatment and long-term survivors), mild COVID-19, anxiety, those with postnatal care needs, and those receiving healthy eating support.

The NHSE/I-commissioned training (initiated in April 2020) recommended that clinically focused VGCs involve at least two members of staff (a facilitator and a consulting clinician). VGCs would start by welcoming patients, confirming their identities, explaining confidentiality, and acquiring verbal or written consent. The session (lasting 1–1.5 hours) would then continue with the clinician consulting individually with each patient in the group setting, drawing on the ‘results board’: usually a PowerPoint slide, shared on the screen for everyone to see, with a table including clinical, self-management and/or quality-of-life information for each patient (depending on condition and review requirements). This was either completed in advance (for example, through blood test results) and/or during the session (for example, using questions such as *‘have your symptoms affected usual activities?’*). In some VGCs on more sensitive topics clinicians decided not to share the board (for example, in postnatal mental health groups). Following individual consultations, the groups usually continued with open discussion and questions on concerns and self-care needs, and sessions ended with patients consolidating learning and personal goals.

Clinical and non-clinical staff who participated in training (roughly two to four staff members per participating practice) described programme support and inter-organisational learning as instrumental:

*‘* [The programme] *has been quite useful because we’ve had training put in place, which has enabled us to quickly train up facilitators that we thought we were going to have to train in-house.’*

(Interview 10, GP)

Finding the ‘right’ VGC format was not without challenges, especially at early stages when practices were experimenting with remote options. Delivery of clinical and mixed formats required a bigger operational and cultural shift from usual care practices; therefore, many practices decided to stay within the relative ‘safety’ of informational or educational sessions.

This study’s longitudinal focus on the two GP sites was mainly driven by how they evolved their group-based care models to deliver primarily clinical (rather than informational or educational) content (see Box 1).

### Relationship-focused care in VGCs

#### Establishing (online) rapport

Staff interviewees discussed VGCs (particularly ones with clinical focus) as having the potential to both strengthen and fragment caring relationships among patients, clinicians, and the practice team. In the early stages, staff worried that their VGCs appeared ‘scripted’ (Interview 18, receptionist/VGC facilitator) and that it would be difficult to establish rapport and patient interaction online compared to face-to-face group consultations:

*‘Normally when you’ve got a* [face-to-face] *group consultation, you work the room, a bit like an actor or a comedian* […] *you kind of get the feel for how is it going, what’s going down well, what isn’t, when are they looking puzzled* […] *it’s really hard to keep talking when you’re not getting any non-verbal feedback.’*

(Interview 2, GP)

Some patients expressed how they were more reticent to share initially, especially when they had faced a significant illness burden (but became more open in subsequent VGCs, having listened to others discussing their personal circumstances). One patient with diabetes (who declined the invitation to join VGCs) explained she did not feel the need to share with a group and saw the process as inefficient, expecting many discussions to be unrelated to her own circumstances:

*‘I’m not really a great joiner-in. I would class myself as not social in that kind of way. I tend to be an individual in terms of getting things done and not wanting to hang out with lots of people, to be honest* […] *those* [the one-to-ones] *I find very efficient. They’re targeted, they’re focused, I get the information that I need and I believe* [the clinicians] *get the information they need.’*

(Interview 26, patient)

#### Relying on pre-existing and new relationships

Having a pre-existing relationship with patients allowed staff to better manage the perceived distance introduced by the online format as they understood how to focus clinically on things that mattered to them. If this relationship was not in place already, clinicians spent time going through medical records for clues on how best to guide the discussion. Where clinicians did not achieve in-depth understanding or when complex individual needs arose in the pandemic, VGCs sometimes led to fragmentation in the care relationship, and the need for additional encounters:

*‘I have an ileostomy, so I was asking some questions and the lady who was running it didn’t really understand what I was trying to ask* […] *it was fine because we did a session separately afterwards which I then sent some information in advance so she could understand … ’*

(Interview 16, patient)

With time, staff found they were better able to facilitate patient relationships and discussion (even if patients had never met each other before, although in a few cases relationships developed between repeat attenders), which increased the value of VGCs:

*‘* […] *now we know what we’re doing; we’ve relaxed a little bit. I feel like that’s obviously come across to the patients as well. So in the last couple it’s been really good patient interaction. There’s been times where we literally can just sit back and just let them there, just having a chat* […] *’*

(Interview 18, receptionist/VGC facilitator)

#### Patients valuing access and connection

The pandemic context appeared to facilitate VGC implementation as many patients and staff became more receptive owing to lockdown and restrictions, disruption to patient support groups, and a shift towards remote interaction: ‘ *it feels much more natural than it would have done before COVID’* (Interview 22, patient). Patients who chose to take part in VGCs preferred the convenience of participating online, particularly as those shielding wanted to avoid the *‘anxiety of being in a public place’* (Interview 21, patient). A patient with mobility issues mentioned how it had been a *‘bit of a nightmare’* getting wheelchair-accessible parking, and that remote participation made her *‘feel more independent’* (Interview 16, patient). Others did not have to take time off work or worry about childcare as they could attend remotely.

One of the patients (a retired health professional) talked about feeling more connected to the practice through engagement in VGCs, as the nurse came to know his situation better and had a more direct relationship with him, rather than only knowing him through his *‘medical records’*, which he described as *‘personal* [but] *not personalised’* (Interview 21, patient). His comments pointed both to an increased level of access and to increased depth in the clinical relationship (*‘it makes it easier and more personal I think that I can phone* [the nurse] *up and she knows’* [Interview 21, patient]), which may, however, become unsustainable if larger numbers of patients participate in VGCs.

Patients also came to value *‘human connection’* (Interview 22, patient) *,* especially when sharing experiences of treatment with peers, and (despite initial reticence from some) they expressed feeling comfortable sharing with others with the same condition:

*‘* [the other patient] *said how much he felt for me because I’d not started my 6 weeks of radiotherapy, and he hoped it would go well. And when I was sort of three-quarters of the way through, he remembered what I was going through and asked me how it was, and that’s a really nice human connection.’*

(Interview 22, patient)

#### Challenges with digital inclusion

It is likely that sessions primarily involved patients with good IT skills. Practices attempted to simplify the remote joining process but there were still patients who faced difficulties, and concerns remained regarding access for those without IT equipment, bandwidth, or confidence to use technology for VGCs.

### Using VGCs to address organisational priorities

#### Staff motivations for setting up VGCs

Some practitioners presented VGC implementation as a next step (albeit not easy) from their face-to-face group consultations programme, as they started to manage the majority of clinical work remotely in COVID-19. Others faced a steeper learning curve as they began remote delivery and group-based care at the same time.

Across practices, motivations for setting up VGCs were primarily demand-led (for example, in a practice so overwhelmed they had adopted a telephone-first triage approach long before the pandemic) and performance-driven (for example, to meet Quality and Outcomes Framework [QOF] requirements for income generation):

*‘I’m hoping that what it will achieve is that we will keep our QOF ticking over. Obviously, that is still a big part of our income and if we took a big hit on QOF then that is very destabilising for the practice.’*

(Interview 2, GP)

Staff interviewees also spoke about setting up VGCs to increase patient access in the context of GP workforce recruitment and retention challenges, support COVID-19 recovery, improve patient satisfaction and experience, enable a ‘coaching’ approach to patient care, achieve better quality clinical consultations, and address isolation in living with chronic illness, compounded by lockdown and the pandemic. Most clinicians and patients suggested that they did not experience an increased need for follow-up after VGCs in a way that would burden the practice.

#### Workload and practice commitment

Many staff participants acknowledged that setting up and delivering VGCs required significant time commitment (about half-day preparation per VGC) and changes to administrative processes. Operational work involved sending invitations and reminders, supporting patients to join the video call, reviewing and updating records, preparing materials, issuing prescriptions, and following up on any individual queries. Introducing this new remote model of group-based care required practice-wide support at all levels, to be able to free up resources and distribute the workload:

*‘* […] *we started where we thought maybe I could do it whilst I was on reception* […] *but we realised rather quickly it’s just not going to work. We needed to have dedicated time* […] *’*

(Interview 18, receptionist/VGC facilitator)

*‘They needed to have at least a champion GP* […] *who could unblock the drain in their practice to get them the resources and time to do it. And the second person who was key was the practice manager, and if both of those people weren’t on board then you could just forget it.’*

(Interview 8, VGC training lead)

#### Enabling relational coordination in practice

VGCs required staff working together or coordinating across rotations to set up and deliver different sessions. In site A the practice pharmacist was supported to different degrees by the nurse manager, healthcare assistant, IT officer, receptionist, medical student, and others. In site B the practice nurse, who had significant autonomy and scope in her role, was supported by a receptionist/VGC facilitator, as well as other NHS and third sector staff (for example, Macmillan nurses and physiotherapists). This need for collaborative practice meant a shift away from traditional hierarchical working and a re-thinking of professional culture in service delivery:

*‘* […] *it’s hard to address those cultural issues and it’s a very different way of working. For the team* [I want to achieve] *better team work, more even stevens, you know, less doctors up here and everyone else down there. So, everyone working together* […] *’*

(Interview 11, GP)

#### Extending roles and skillset on remote group-based care

Enthusiastic staff (clinical and non-clinical) were prepared to make significant effort so that VGCs would work in their practice, and found the experience rewarding. Some suggested that they extended their skillset and were able to take on additional leadership, clinical, or operational responsibilities, such as IT staff taking front-end roles to support patients, receptionists delivering group facilitation, and healthcare assistants learning about diabetes:

*‘They’ve learnt an awful lot the two healthcare assistants who are doing this; they’ve learnt a lot about diabetes.* […] *We’ve learnt a lot. I learn from the patients because we’re a very multi-ethnic area. I learn an awful lot about things from patients and some of it I’m quite shocked about.’*

(Interview 25, nurse manager)

Other staff, however, seemed more reticent to engage with this model, including those who received VGC training but later refrained from taking on any VGC responsibilities. This was primarily because of a lack of time and organisational slack, and the complexity of group-based care:

*‘So, we have members of the team that have been trained and they’re taking it in turns* […] *everybody’s rotating, so it’s not too much work for one person. There has* [sic] *been a few emails back saying, “I haven’t got time for this,” and so on* […] *it’s all very time consuming.’*

(Interview 10, GP)

#### IT and infrastructure challenges

Supporting patients to access the video platform involved significant background work, as one of the receptionists explained: *‘patients that need a little bit of help, just give them a call and just talk them through how to just to get onto the meeting and things like that’* (Interview 18, receptionist).

Early in the pandemic, some practices faced IT equipment shortage, which meant not all staff could access webcams. Network bandwidth was a barrier to remote care in some practices (although this improved with time).

The video platform used to deliver VGCs (MS Teams) brought challenges as some of its features and integration with practice systems were difficult to configure and changed over time. Staff reflected that such technical problems looked *‘unprofessional’* (Interview 10, GP), and could influence patient engagement with group-based care:

*‘* […] *a lot of our patients said that they didn’t receive the email. And some of the patients were in the lobby and they couldn’t get in* […] *some people weren’t getting a chat box, so they had to keep going out and trying back in again* […] *that looked a bit unprofessional at the beginning* […] *’*

(Interview 10, GP)

**Figure 2.**
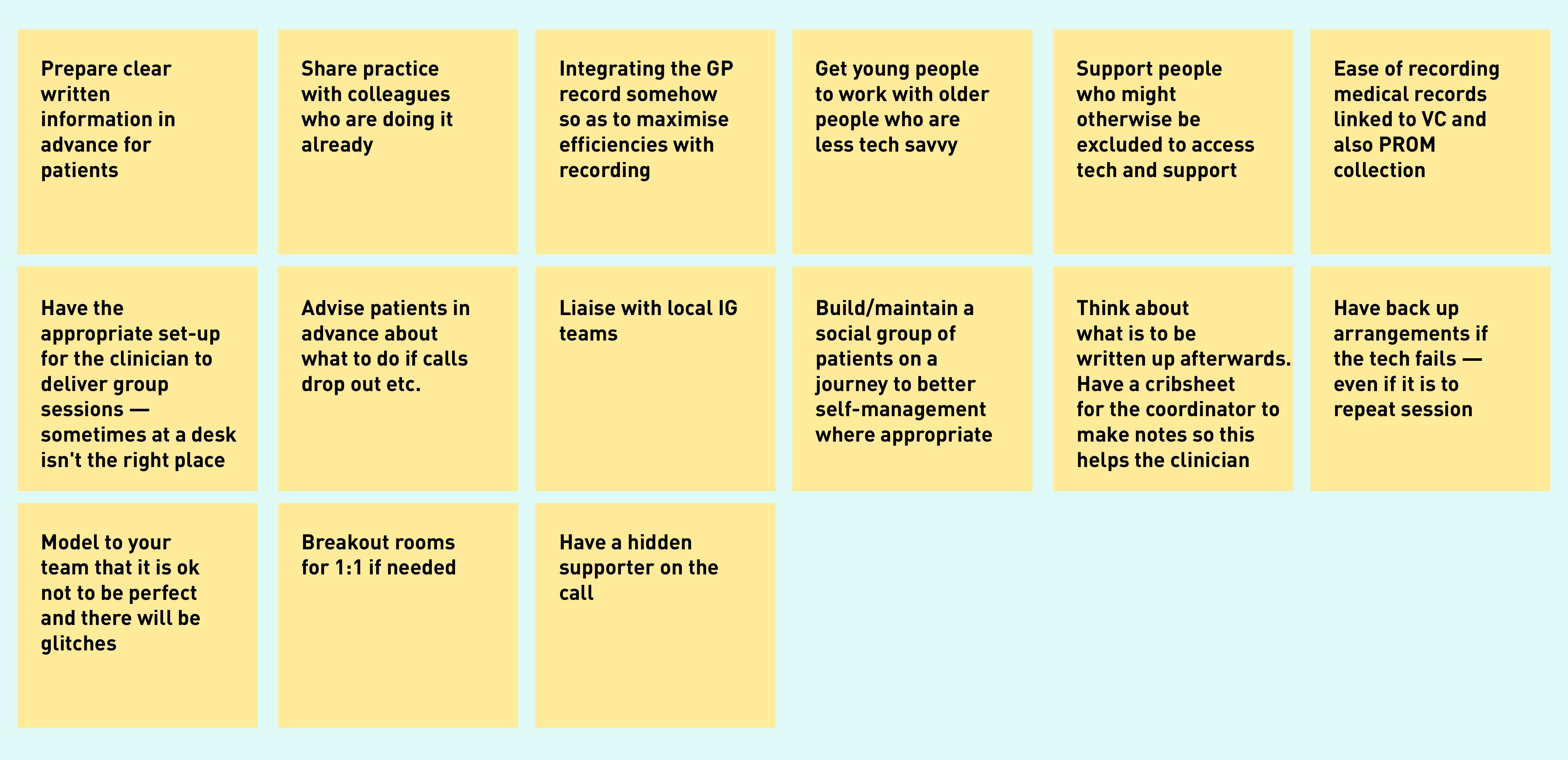
*Post-it notes by participants in co-design workshops presenting ideas on improving video group consultation delivery. IG = information governance. PROM = Patient Reported Outcome Measures. VC = video consultation.*

In co-design workshops, participants shared several ideas on improving technology-supported group-based care, including on the physical space and recording systems required at practice level, best approach to managing technical glitches and lack of digital confidence, and support required at the patient end (Figure 2).

#### Balancing concerns on patient risk and information governance

Governance around remote care was partly relaxed in the COVID-19 context. However, VGCs raised new ethical concerns around online confidentiality, consent, and risk that the national VGC programme sought to address. As this resulted in a short pause to programme delivery, some clinicians made their own local clinical safety judgments, balancing concerns about confidentiality with the need to address safeguarding issues in their locality:

*‘* […] *we’ve taken the decision not to pause because we have indemnity cover from NHS, well we have from the MPS* [Medical Protection Society]. *And also the other decision that we didn’t pause is because when we had safeguarding issues in our area; we have shaken babies.’*

(Interview 10, GP)

## DISCUSSION

### Summary

This study examined how remote group-based care was implemented in English general practice during the first year of the COVID-19 pandemic (2020–2021). Findings show that VGCs were organised in different ways depending on practice-level needs and priorities and organisational capacity for innovation (including experience with face-to-face group consultations), and incorporated different degrees of clinical, educational, and/or informational content.

The majority of patients valued the human connection with peers and increased access and engagement with their practice via group-based care. VGC implementation (especially clinical formats) required changes in operational processes, shifts in professional roles, increased collaborative working and staff capacity, digital and online facilitation skills, and availability of equipment and IT infrastructures.

Staff required training and support with this complex change effort, delivered under extreme circumstances during the pandemic and with increased awareness of risk. Staff and patients were supportive of VGCs continuing beyond the crisis context.

### Strengths and limitations

To the authors’ knowledge, this is the first study on VGCs in English general practice. Data were collected across eight practices and focused on two sites for a more longitudinal view. Underpinned by a robust theoretical framework, the analysis highlights a number of interacting influences shaping implementation of remote group-based care in general practice. Given the small sample, the analysis may have missed wider developments with VGCs elsewhere. The research would have been strengthened by observation in group sessions and additional patient interviews, especially with those not taking part in VGCs.

### Comparison with existing literature

To the authors’ knowledge, only two small-scale pilot studies have been published on VGCs so far (in secondary care in the UK and in a community-based outpatient diabetes clinic in rural Guam), reporting improvement in some patient and service outcomes.^30^^,^^31^ A larger body of work on face-to-face group consultations corroborates improvements in biomedical outcomes and patient satisfaction for those participating in groups across a range of conditions and settings.^15^^,^^32^^–^^34^ The patient interviewees in the present study had largely positive experiences, but a few challenged the value of remote group-based care, owing to concerns about digital inclusion and distraction from the clinician–patient relationship.

It is a prerequisite for group consultations to incorporate clinical care in a group setting (rather than purely education or peer support). The extent to which this happens and the format it takes varies. Different models have been adopted to meet local needs and priorities, with remote and face-to-face approaches developing over time, and calls to action promoting further implementation in different contexts.^32^^,^^33^^,^^35^^,^^36^ Instead of a single standardised format, the literature proposes common organising principles for successful delivery of VGCs (and technology-supported care in general), including attention to the role of patients as active co-production partners, and reconfiguration of care delivery so that VGCs provide added value.^13^^,^^37^

VGCs are frequently promoted as being cost-cutting and time-saving. There are currently little data to support this, especially in the short term.^12^^,^^32^ Several practice-level and system-level challenges have been documented in general practice and beyond.^12^^,^^19^^,^^35^^,^^36^ These include lack of organisational buy-in, barriers owing to hierarchical working, significant time commitment needed to set up and deliver group-based care, and resource limitations.^12^^,^^33^^,^^35^

Previous studies (including one by the authors of the present work) have highlighted the importance of facilitation skills (and training) to support meaningful patient participation.^12^^,^^19^ This research adds to this literature by highlighting the additional complexity remote group-based care introduces, in terms of IT infrastructure and equipment, remote facilitation and digital skills (different to those needed for in-person group consulting), online confidentiality and indemnity concerns, extra staff needed to run remote sessions, and IT support for patients. Video and (pre-pandemic) in-person group consulting have been deployed in general practice to meet performance targets and sustain income in the context of already unrealistic workloads. Yet, it is unclear how growing NHS pressures will influence the role group consulting may play in the ongoing recovery process. In-depth research and knowledge-sharing about this model will be critical to supporting appropriate use and roll-out in different settings.

### Implications for research and practice

Despite implementation challenges, VGCs afford opportunities for increased access to peer-focused clinical care, otherwise not available face-to-face or in individual appointments. However, with VGCs in their infancy and involving small numbers of patients, it is too early to draw conclusions on the extent to which they can be meaningfully embedded in routine practice. Further mixed-methods research is needed to understand how VGCs might provide value and contribute to re-configuration of care towards a more affordable, sustainable, and patient-centred model. There is still much to examine, especially when considering how remote care is evolving in a world less concerned with physical distancing.

System support is needed to facilitate adoption of this new model of care at scale, including funding, access to training, video platforms that are integrated with wider operational work at practice level, and digital inclusion initiatives. Future evaluation must extend beyond early enthusiastic innovators.
